# Medicaid Expansion and Overall Survival of Lower Gastrointestinal Cancer Patients After Cytoreductive Surgery and Heated Intraperitoneal Chemotherapy

**DOI:** 10.1245/s10434-024-16446-8

**Published:** 2024-11-15

**Authors:** Kirithiga Ramalingam, Liang Ji, Michael P. O’Leary, Sharon S. Lum, David Caba Molina

**Affiliations:** 1 Department of Surgery, University of California San Francisco, San Francisco, CA USA; 2 School of Public Health, Loma Linda University, Loma Linda, CA USA; 3 Department of Surgical Oncology, University of California Irvine, Irvine, CA USA; 4 Department of Surgery, Loma Linda University, Loma Linda, CA USA; 5 Loma Linda University, Loma Linda, CA USA; 6 Riverside University Health System Medical Center, Moreno Valley, CA USA

## Abstract

**Background:**

In the United States, often only tertiary centers offer cytoreductive surgery and heated intraperitoneal chemotherapy (CRS+HIPEC) for peritoneal metastases in advanced lower gastrointestinal malignancies. Growing evidence shows that Medicaid expansion under the Affordable Care Act (ACA) of 2010 enhanced healthcare access and outcomes.

**Objective:**

We sought to determine whether Medicaid expansion was associated with decreased all-cause mortality of lower gastrointestinal cancer patients following CRS+HIPEC.

**Methods:**

We analyzed data from the National Cancer Database (2010–2019) on lower gastrointestinal cancer patients who underwent CRS+HIPEC. Medicaid expansion, introduced under the ACA in 2010, extends health insurance to low-income adults. We categorized states by expansion timing: early (2010–2013), immediate (January 2014), late (after January 2014), or no expansion to assess the impact of Medicaid expansion on mortality using a multivariable Cox regression model.

**Results:**

Of the 1001 study patients, 671 (67%) were diagnosed in Medicaid expansion states. Grade and Medicaid expansion status were the only factors independently associated with overall survival on multivariable analysis. On average, patients in Medicaid expansion states experienced a 4% increase in annual survival compared with those in non-expansion states who had a 1% decrease in annual survival over the study period.

**Conclusions:**

Patients from states that had an early expansion of Medicaid and patients with lower-grade tumors had significantly better overall survival. Our study findings suggest that improved access to healthcare through Medicaid expansion was associated with increased survival rates of lower gastrointestinal cancer patients who undergo CRS+HIPEC for the treatment of peritoneal metastases.

In the United States (US), colorectal cancer is the third most common cancer diagnosed in males and females, and the second leading cause of cancer-related deaths overall.^1^ The relative incidence of synchronous peritoneal metastases in colorectal cancer is 4–15%. Approximately 8% of patients with primary resection for colorectal cancer and up to 25% of those with recurrent colorectal cancer will develop peritoneal metastatic disease.^2–4^ Advanced appendiceal and colorectal malignancies with peritoneal involvement may be treated with multimodal therapy, including cytoreductive surgery and heated intraperitoneal chemotherapy (CRS+HIPEC),^5^ which has been associated with better survival for select populations.^6–8^ With many centers providing training, CRS+HIPEC has become increasingly available in the US.

Age, sex, race, ethnicity, socioeconomic status, comorbidity index, stage, and grade have been associated with disparities in survival outcomes in previous studies of colorectal cancer patients.^9–12^ Disparities in survival outcomes of colorectal cancer patients related to insurance status have also been well-documented.^9,13,14^ There are limited and conflicting studies regarding the association of insurance status with outcomes of patients receiving CRS+HIPEC;^15,16^ hence, it is essential to identify the knowledge gap in this particular population to address and achieve equity in cancer care.

Medicaid was established in the US as a joint federal-state government initiative to cover the costs of providing healthcare to people who do not have the financial means to buy health insurance. The Patient Protection and Affordable Care Act (ACA) was passed in April 2010 with the goal of expanding Medicaid eligibility to include all adults in the US under the age of 65 years with incomes below 133% of the federal poverty threshold. However, in 2012, the Supreme Court ruled that the decision to implement the proposed expansion should be made at the individual state level. This led to some states having expanded access while others have yet to do so.^17^ Medicaid expansion has considerably reduced under- and uninsured rates, improving access to healthcare and clinical outcomes.^18–20^ We hypothesized that lower gastrointestinal patients in Medicaid expansion states have decreased all-cause mortality, i.e., better overall survival (OS) than those in Medicaid non-expansion states following CRS+HIPEC for treating peritoneal metastasis secondary to improved access to healthcare.

## Methods

We performed a retrospective study of the prospectively maintained National Cancer Database^21^ (NCDB) to identify colorectal and appendiceal cancer patients who received CRS+HIPEC for the treatment of peritoneal metastases between 2010 and 2019 (*n* = 1,686,787). The NCDB is a joint project of the Commission on Cancer of the American College of Surgeons and the American Cancer Society. The data used in the study are derived from a de-identified NCDB file. The American College of Surgeons and Commission on Cancer have not verified and are not responsible for the analytic or statistical methodology employed or the conclusions drawn from these data by the investigator.^21^

**Fig. 1:**
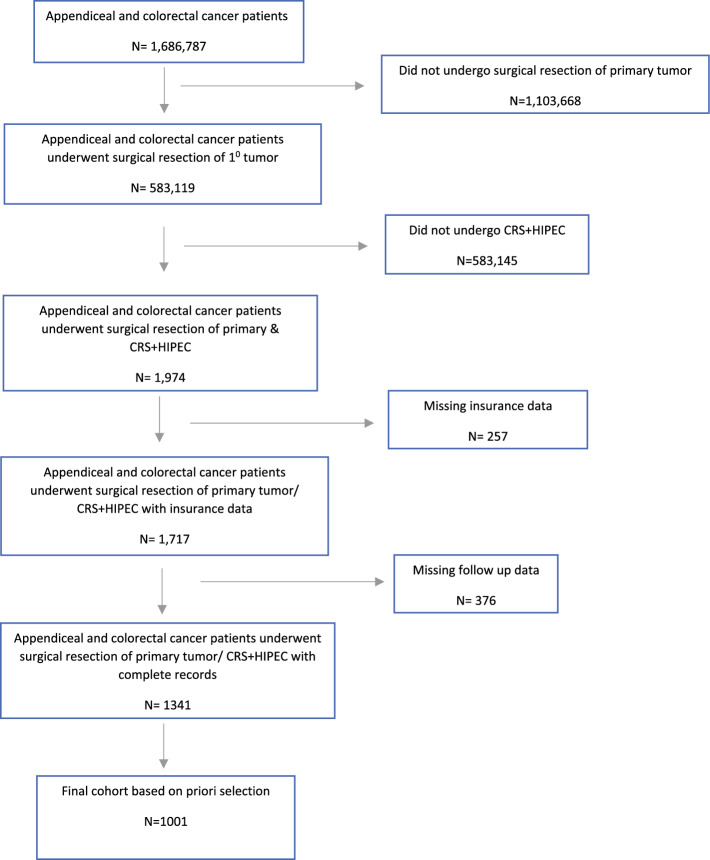
Patient selection process. *CRS* cytoreductive surgery, *HIPEC* heated intraperitoneal chemotherapy

We selected surgically treated colorectal and appendiceal cancer patients with the following surgical codes: colon: 30, 31, 40, 50, 51, 55, 56, 57, 60, 65, 66, 70, 80; rectosigmoid: 60, 70, 80; and rectum: 30, 40, 50. To identify the study population of colorectal and appendiceal cancer patients with peritoneal metastasis who had undergone CRS+HIPEC during the relevant time period, we used the following systemic/surgery sequence codes to identify patients who had undergone CRS+HIPEC: intraoperative systemic therapy: 5; and intraoperative systemic therapy with other systemic therapy administered before or after surgery: 6. Patients who did not have details on insurance status. Follow-up, income, education, race and ethnicity, and grade were excluded. Patients under 40 years of age were, by default, excluded from the study as Medicaid expansion status is only determined in the NCDB for patients over 40 years of age. Medicaid expansion status of states in which patients were diagnosed were classified into the following categories by the NCDB: Medicaid non-expansion states, i.e. states that did expand Medicaid past 2014: TN, NC, ID, GA, FL, MO, AL, MS, KS, TX, WI, UT, SC, SD, VA, OK, NE, WY, and ME; early-expansion states, i.e. states that expanded Medicaid between 2010 and 2013: WA, CA, NJ, MN, DC, and CT; January 2014 expansion states: KY, NV, CO, OR, NM, WV, AR, RI, AZ, MD, MA, ND, OH, IA, IL, VT, HI, NY, and DE; and late-expansion states (after January 2014): NH, IN, MI, PA, AK, MT, LA.^22^ The case selection schema is shown in Fig. 1.

Statistical analyses were conducted using SAS version 9.4 (SAS Institute, Inc., Cary, NC, USA). The primary exposure variable studied was the Medicaid expansion status of states in which patients were diagnosed, while the outcome variable studied was the OS, which was defined by the length of time between the date of diagnosis and the date of death or the last documented follow-up in the NCDB. Descriptive statistics were used to investigate the sociodemographic and clinical characteristics of the study population, and the Chi-square test was used to evaluate differences between Medicaid expansion and Medicaid non-expansion groups. A multivariable Cox proportional hazard regression model was performed using the *a priori* selection method to study the association between the Medicaid expansion status of states in which patients were diagnosed (non-expansion, early expansion, January 2014 expansion, and late expansion) and all-cause mortality after adjusting for age at diagnosis (40–65 years and >65 years), sex (male, female), race and ethnicity (non-Hispanic Black, non-Hispanic White, Hispanic, Asian/other), insurance status (uninsured, Medicaid, Medicare, privately insured, other government), program type (community, comprehensive community, academic/research, integrated network), year of diagnosis, grade (well-differentiated, moderately differentiated, and poorly differentiated), and Charlson–Deyo comorbidity score (0,1,2,3). Income and education variables were removed from the multivariable model since the proportion of missing values was >20% of the final study cohort (21.9% and 21.7%, respectively). Univariate Cox proportional hazard regression analysis was performed to assess the annual survival trend (2010–2017) in Medicaid expansion and non-expansion states. We chose 2017 as the upper limit for survival assessment, given that the median survival in this study population was 22–41 months.

## Results

Of the 1001 patients, 671 (67%) were from the Medicaid expansion states and 330 (33%) were from the Medicaid non-expansion states. The majority (75.8%, *n* = 759) of study patients were younger than 65 years of age and had appendiceal primary (77%, *n* = 771). The majority of patients were of non-Hispanic White race and ethnicity (81.7%, *n* = 818) and had well-to-moderately differentiated tumors (75.3%, *n* = 754). Insurance status included 628 (62.7%) privately insured and 265 (26.5%) Medicare, 65 (6.5%) Medicaid, 24 (2.4%) other government insurance, and 19 (1.9%) uninsured. Medicaid expansion had a significantly higher proportion of Hispanic and Asian/other patients (10.3 vs. 5.2%; Chi-square test, *p* = 0.006), patients from residential areas with a higher median income (49.6 vs. 33%; Chi-square test, *p ≤* 0.001), patients with Medicaid insurance (8.1% vs. 3.3%; Chi-square test, *p* < 0.001), and lower proportion of uninsured (1.6% vs. 2.4%; Chi-square test, *p* < 0.001) when compared with the Medicaid non-expansion group (Table 1). The median follow-up time was 53 and 48.5 months for the Medicaid non-expansion and expansion groups, respectively, and the median survival time for the Medicaid non-expansion and expansion groups was 93.8 and 107 months, respectively.

**Table 1:** Sociodemographic and clinical characteristics of the study population by Medicaid expansion status

	Overall [*N* = 1001]		No Medicaid expansion [*n *= 330 (33%)]		Medicaid expansion [*n *= 671 (67%)]		*p* value^a^
	*N*	%	*n*	%	*n*	%	
Age, years							
40–65	759	75.8	254.0	77.0	505.0	75.3	0.553
Age >65	242	24.2	76.0	23.0	166.0	24.7	
Sex							
Male	480	48.0	156.0	47.3	324.0	48.3	0.763
Female	521	52.1	174.0	52.7	347.0	51.7	
Race and ethnicity							
Non-Hispanic White	818	81.7	273.0	82.7	545.0	81.2	0.006
Non-Hispanic Black	97	9.7	40.0	12.1	57.0	8.5	
Hispanic	48	4.8	13.0	3.9	35.0	5.2	
Asian/other	38	3.8	4.0	1.2	34.0	5.1	
Income (residence area median income), missing = 219							
< $40,227	119	15.2	63.0	22.8	56.0	11.1	<0.001
$40,227–50,353	141	18.0	62.0	22.5	79.0	15.6	
$50,354–63,332	180	23.0	60.0	21.7	120.0	23.7	
> $63,332	342	43.7	91.0	33.0	251.0	49.6	
Education (residence area high-school graduation failure proportion), missing = 217							
≥ 17.6%	120	15.3	46.0	16.7	74.0	14.6	0.148
10.9–17.5%	179	22.8	73.0	26.5	106.0	20.9	
6.3–10.8%	246	31.4	84.0	30.4	162.0	31.9	
< 6.3%	239	30.5	73.0	26.5	166.0	32.7	
Insurance status							
Medicaid	65	6.5	11.0	3.3	54.0	8.1	<0.001
Medicare	265	26.5	85.0	25.8	180.0	26.8	
Privately insured	628	62.7	210.0	63.6	418.0	62.3	
Uninsured	19	1.9	8.0	2.4	11.0	1.6	
Other government insurance	24	2.4	16.0	4.9	8.0	1.2	
Program							
Community Cancer Program	200	20.0	54.0	16.4	146.0	21.8	0.121
Academic/Research Program	664	66.3	231.0	70.0	433.0	64.5	
Integrated Network Cancer Program	137	13.7	45.0	13.6	92.0	13.7	
Charlson–Deyo comorbidity							
0	823	82.2	268.0	81.2	555.0	82.7	0.947
1	140	14.0	49.0	14.9	91.0	13.6	
2	21	2.1	7.0	2.1	14.0	2.1	
3	17	1.7	6.0	1.8	11.0	1.6	
Grade							
Well-differentiated	374	37.4	138.0	41.8	236.0	35.2	0.043
Moderately differentiated	380	38.0	108.0	32.7	272.0	40.5	
Poorly differentiated	247	24.7	84.0	25.5	163.0	24.3	

^a^Chi-square test *p* value

In the multivariable Cox regression model, the grade of the disease and being diagnosed in Medicaid early-expansion states were found to be independently associated with decreased hazard of death, i.e., all-cause mortality (increased OS). Hazard of death (all-cause mortality) in patients with moderately and poorly differentiated tumors were 2.1 and 4.7 times higher, respectively, than in those with well-differentiated tumors (*p* < 0.001). It is important to note that the hazard of death was significantly lower in patients from the early-expansion states compared with those from the non-expansion states (hazard ratio (HR) 0.71, 95% confidence interval (CI) 0.52–0.97, *p* = 0.029). Age, sex, race and ethnicity, insurance type, program type, year of diagnosis, and comorbidity index were not independently associated with all-cause mortality (Table 2).

**Table 2:** Multivariable Cox regression analysis model for all-cause mortality (*n* = 939)

	HR	95% CI		*p* value
		Lower limit	Upper limit	
Age, years				
40–64	1.06	0.76	1.46	0.749
65+	Ref			
Sex				
Female	0.88	0.72	1.09	0.245
Male	Ref			
Race/ethnicity				
Asian/other	0.78	0.44	1.38	0.395
Non-Hispanic Black	1.14	0.81	1.60	0.447
Hispanic	1.06	0.63	1.80	0.817
Non-Hispanic White	Ref			
Grade				
Well-differentiated	Ref			
Moderately differentiated	2.12	1.61	2.78	<0.001
Poorly/undifferentiated	4.71	3.59	6.18	<0.001
Insurance				
Medicaid	0.76	0.48	1.20	0.234
Medicare	1.24	0.91	1.68	0.174
Other Government	1.04	0.58	1.86	0.897
Not insured	0.76	0.33	1.72	0.504
Private insurance	Ref			
Medicaid expansion status				
Early-expansion states	0.71	0.52	0.97	0.029
January 2014 expansion states	0.90	0.70	1.15	0.399
Late-expansion states	1.03	0.76	1.39	0.845
Non-expansion states	Ref			
Charlson–Deyo score				
0	Ref			
1	1.19	0.89	1.58	0.236
2	0.89	0.41	1.92	0.766
3	1.80	0.92	3.53	0.086
Program				
Academic/Research	0.94	0.73	1.21	0.630
Integrated Network	1.07	0.76	1.51	0.700
Community Cancer Program	Ref			
Year of diagnosis				
Annual	0.97	0.93	1.02	0.184

*HR* hazard ratio, *CI* confidence interval, *Ref* reference

**Fig. 2:**
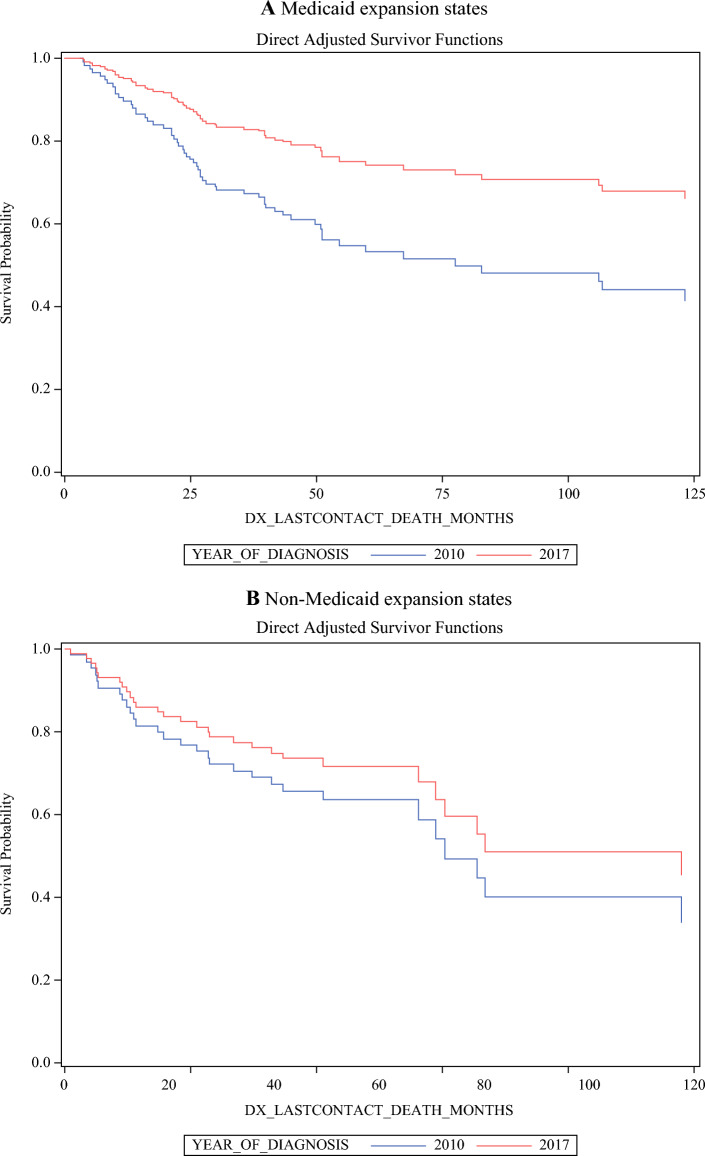
Trend of overall survival in (**A**) Medicaid expansion states versus (**B**) non-expansion states

When analyzing the trend of OS based on the year of diagnosis (2010–2019) using a univariate Cox regression model, the Medicaid non-expansion states had a 1% annual OS decrement (HR 1.011, 95% CI 0.94–1.09, *p* = 0.773), whereas Medicaid expansion states had a 4% annual survival improvement (HR 0.96, 95% CI 0.91–1.01, *p* = 0.123). The above improvement in the annual trend of OS is shown in Fig. 2, which compares the survival curves of patients diagnosed in 2010 and 2017 between the Medicaid expansion and non-expansion groups.

## Discussion

This study investigated the association between state Medicaid expansion status and survival outcomes in patients undergoing CRS+HIPEC for colorectal and appendiceal malignancies. We found that Medicaid early-expansion status and grade are independent predictors of survival. Early adopters of Medicaid expansion often face unique challenges and opportunities, leading to innovative practices or policy adjustments that may not be immediately replicated by late adopters. Changes in the Medicaid expansion program over time may also influence the outcomes observed in late-expansion states. Furthermore, compared with those without Medicaid expansion, year-over-year survival improvements were observed in patients with colorectal and appendiceal malignancies treated with CRS+ HIPEC in states with Medicaid expansion. These findings suggest that Medicaid expansion has improved survival outcomes likely by improving access to healthcare and narrowing the equity gap in cancer care and outcomes. Our data on travel distances provide compelling evidence supporting the improved access to healthcare in Medicaid expansion states. Patients in Medicaid expansion states had a significantly lower median travel distance to the hospital {19.3 miles (interquartile range [IQR] 51.7 miles) vs. 39.95 miles (IQR 114.6 miles), *p* < 0.0001}.

Compared with prior studies, the main strength of this work is its large sample size for a rare patient population. NCDB captures approximately 72% of newly diagnosed cancer cases in the US.^21^ Previous studies examining the relationship between insurance status and survival in this patient population were limited by small sample sizes, did not specifically focus on gastrointestinal malignancies, and yielded conflicting results.^15,16^

One single-institution study with 31 patients found no impact of insurance on the survival of patients who underwent CRS+HIPEC for various primary and secondary peritoneal carcinomatoses.^15^ Another retrospective cohort study, also from a single institution, showed lower OS in the underinsured group (including Medicaid and uninsured patients) compared with the privately insured group in patients who received CRS+HIPEC for peritoneal carcinomatosis.^16^

While prior studies have demonstrated disparities in outcomes for CRS+HIPEC in various patient populations, this study found no association between age, sex, race and ethnicity, program type, comorbidities, and survival in patients undergoing CRS+HIPEC. It is worth mentioning that we did not find an association between the female sex and improved survival as compared with a study focused solely on appendiceal malignancy patients who underwent CRS+HIPEC.^23^ This is likely due to the difference in the study cohort, i.e., our study included both colorectal and appendiceal malignancies, unlike the other study that included only appendiceal malignancies.

Several limitations of this study should be acknowledged. The need for specific procedural codes for CRS in the NCDB could impact the accuracy of the analysis. However, our method has been standardized with concurrent treatment regimens only particular to CRS+HIPEC. Disease-specific survival data are not available in the NCDB; therefore, our analysis relies on OS as the primary outcome measure. Incomplete data and loss of follow-up are inherent limitations of retrospective analyses and could introduce bias. The NCDB collects data from the Commission on Cancer-accredited programs, potentially limiting generalizability to patients treated outside these centers.

The implications of these findings are significant for public health and clinical medicine. The study provides evidence that Medicaid expansion is associated with improved survival outcomes in patients undergoing CRS+HIPEC for colorectal and appendiceal malignancies.

Additional studies should address the limitations identified in this study. Institutional or registry studies may allow for the collection of additional data, such as the Peritoneal Carcinomatosis Index (PCI), which is considered an essential prognostic factor in patients undergoing CRS+HIPEC. Additionally, specific procedural codes for CRS and HIPEC, as well as disease-specific survival, would enable more accurate analysis and interpretation.^24,25^ Further research is also warranted to explore the impact of insurance status on survival outcomes in other cancer types and patient populations.

## Conclusion

Our study demonstrates a significant association between residing in a state with Medicaid expansion and improved OS for patients undergoing CRS+HIPEC for lower gastrointestinal cancer with peritoneal metastases. This finding suggests that increased access to healthcare, potentially facilitated by Medicaid expansion, may be a contributing factor to improved patient outcomes.
